# Elasticity and substitutability of food demand and emerging disease risk on livestock farms

**DOI:** 10.1098/rsos.221304

**Published:** 2023-03-15

**Authors:** Alexis Delabouglise, Guillaume Fournié, Marisa Peyre, Nicolas Antoine-Moussiaux, Maciej F. Boni

**Affiliations:** ^1^ CIRAD, UMR ASTRE, Montpellier 34398, France; ^2^ UMR ASTRE, University of Montpellier, CIRAD, INRAE, Montpellier, France; ^3^ Department of Pathobiology and Population Sciences, Royal Veterinary College, Veterinary Epidemiology, Economics and Public Health Group, University of London, Hawkshead Lane, Hatfield, Hertfordshire AL97TA, UK; ^4^ Universitá de Lyon, INRAE, VetAgro Sup, UMR EPIA, Marcy l'Etoile, France; ^5^ Université Clermont Auvergne, INRAE, VetAgro Sup, UMR EPIA, Saint Genes Champanelle, France; ^6^ FARAH-Fundamental and Applied Research for Animals and Health, University of Liège, Avenue de Cureghem 7A-7D, Liège 4000, Belgium; ^7^ Department of Biology, Center for Infectious Disease Dynamics, Pennsylvania State University, University Park, PA 16802, USA

**Keywords:** health economics, veterinary epidemiology, livestock production, food safety, game theory, price theory

## Abstract

Disease emergence in livestock is a product of environment, epidemiology and economic forces. The environmental factors contributing to novel pathogen emergence in humans have been studied extensively, but the two-way relationship between farm microeconomics and outbreak risk has received comparably little attention. We introduce a game-theoretic model where farmers produce and sell two goods, one of which (e.g. pigs, poultry) is susceptible to infection by a pathogen. We model market and epidemiological effects at both the individual farm level and the community level. We find that in the case of low demand elasticity for livestock meat, the presence of an animal pathogen causing production losses can lead to a bistable system where two outcomes are possible: (i) successful disease control or (ii) maintained disease circulation, where farmers slaughter their animals at a low rate, face substantial production losses, but maintain large herds because of the appeal of high meat prices. Our observations point to the potentially critical effect of price elasticity of demand for livestock products on the success or failure of livestock disease control policies. We show the potential epidemiological benefits of (i) policies aimed at stabilizing livestock product prices, (ii) subsidies for alternative agricultural activities during epidemics, and (iii) diversifying agricultural production and sources of proteins available to consumers.

## 1. Introduction

Livestock farming systems have played a major historical role in the evolution and establishment of major human pathogens [1] and are still a predominant driver of pathogen emergence in humans [2]. Additionally, certain livestock pathogens having, at the moment, no or very limited pathogenicity in humans, such as peste des petits ruminants (PPR) and Newcastle disease, are a continuous threat to people’s livelihoods in lower- and middle-income countries (LMIC) in Africa and Asia, and they undermine the efficiency of our use of environmental resources to cover the protein needs of an increasingly larger human population [3]. Other infectious diseases such as African swine fever (ASF), which were previously circumscribed to restricted areas, are now propagating to a large number of countries causing major supply shocks and threatening the livelihood of low-income populations [4]. Difficulties in identifying and implementing effective disease control programmes have raised questions of the added value of policy optimization based on an economic rationale [5,6]. To this end, it is necessary to incorporate the strategic responses of actors of animal production into epidemiological models.

While the impact of strategic behaviour of economic agents in the face of epidemics has attracted significant attention over the past two decades [7], few studies have addressed the specific issue of behavioural responses of livestock farmers to the spread of diseases in livestock populations and their market effects. The incentivization of ex ante disease prevention through enhanced farm biosecurity has been the focus of most theoretical behavioural studies so far, with recommendations mostly tailored to the livestock sector of industrialized countries [5,8–10].

Disease risk due to poor hygiene occurs primarily in LMICs where livestock producers lack access to financial capital and technology, which limits their capacity to invest in infrastructure or veterinary interventions for disease prevention [5,11]. A majority of smallholder farmers in LMICs rely on a low input production system based on free grazing or the use of swill for animal feeding, which is incompatible with most biosecurity requirements [12]. Free-grazing animals from different farms frequently intermix and are exposed to contaminated material present in the environment and to frequent contacts with wildlife [13]. Vaccination constitutes a key preventive response, but depending on the disease and the socio-economic context vaccines against the considered pathogen may simply not exist—this is the case for ASF [14]—or their delivery to farmers may be impaired by constraints in production, transport and storage [15]. Surveys in LMICs show a strong positive correlation between herd size and vaccine uptake [16,17], suggesting that vaccination is more likely to be adopted in large-scale farming systems; however, the vast majority of livestock producers are still smallholders.

The appeal of smallholder farming partly comes from its diversification of income sources, a major risk-coping strategy of rural households [18]. Keeping livestock on a limited scale or with low fixed investments in building or equipment allows farmers to easily adapt their production size or temporarily empty their farm to invest in other economic activities, in response to epidemics or market shocks. As a consequence, farmers may react to epidemics affecting either market prices or their productivity by modulating their flock/herd sizes or switching focus to a different species—rather than investing in prevention. The frequent occurrence of species changes in farmed livestock at the household level, in low- and middle-income countries, is supported by empirical observations detailed in electronic supplementary material, S1. Socio-economic surveys conducted in northern Vietnam have revealed that commercial pig farmers react to epidemics of classical swine fever or porcine reproductive and respiratory syndrome by expanding their stock of breeding sows, rather than slaughtering them, in order to produce a larger pool of fattened pigs in anticipation of production shortage and high prices [19]. Broiler chicken farmers employ both strategies, most of them reducing their stock of day-old chicks upon being informed of an epidemic, while some of them do the exact opposite, as they aim at being ready to sell adult chickens during price peaks following epidemics [20]. These empirical observations underline the need of new modelling approaches incorporating farmers’ responses to variations in both disease risk and prices.

Epidemics of livestock diseases affect markets through supply and demand shocks [21]. Supply shocks originate from livestock product shortage due to a drop in production caused by (i) the disease itself, (ii) policies implemented to control the epidemics, or (iii) the downsizing of farm production by farmers out of fear. Demand shocks are due to information obtained by consumers on disease occurrence in the livestock population leading them to decrease their demand for products of the affected species [22,23]. Empirical studies showed that consumers’ demand for animal products quickly adapts to information received on health hazards, although the magnitude of changes in the concerned products’ demand varies according to the pathogen and sociocultural context [24,25]. Depending on the relative intensity of these two shocks, market prices may evolve in opposite ways (some examples are detailed in electronic supplementary material, S2).

The present study aims at providing a theoretical framework for the modelling of the joint dynamics of livestock pathogens and livestock markets when farmers change their herd size or species in response to changes in market prices, perceived risk of mortality or mandated depopulation. We determine how elasticity of demand for livestock meat, the substitutability between food products, and consumer preference for healthy animals can influence the outcome of disease control policies and suggest potential directions for improvement of these policies.

## 2. Model

### 2.1. Description of the system

We model a system composed of a population of households practising the breeding and fattening of animals of a given species for the purpose of meat production in a given region. The breeding-fattening model applies to most ruminant and pig farms and to smallholder chicken farms where farmers aim at producing mature animals eligible for slaughter and consumption while keeping some adults as breeders to ensure self-renewal of their herd or flock. Each farmer keeps one herd and aims at optimizing herd size and sell rate. Similar to the model proposed by Nerlove & Bessler [26], we consider that herds are entirely self-renewed, i.e. departing animals are replaced through the breeding of adult animals present in the herd and not from the purchase of animals from outside. This assumption is consistent with a several demographic and economic models of small-scale poultry or extensive ruminant enterprises in low- or middle-income countries, where introduction of animals from outside play a very minor role in the herd turnover [27,28]. Farmers fatten and sell their surplus animals, i.e. the difference between the number of newborn animals and the number of replacement animals needed to renew the breeding herd. Households have multiple activities and are simultaneously engaged in the supply of an alternative good. The two goods are assumed to be perfect substitute-in-production, meaning that their production consumes the same resources and animal production is assigned an opportunity cost in our model. Animals of the considered species are susceptible to an infectious disease, which is transmitted through direct contact between infected and susceptible animals. This disease does not affect the alternative good. We only consider the transmission of the disease between domestic animals in this model and do not explicitly model its risk of introduction from an external source of infection, including wildlife.

### 2.2. Farm economic model

**Figure 1.**
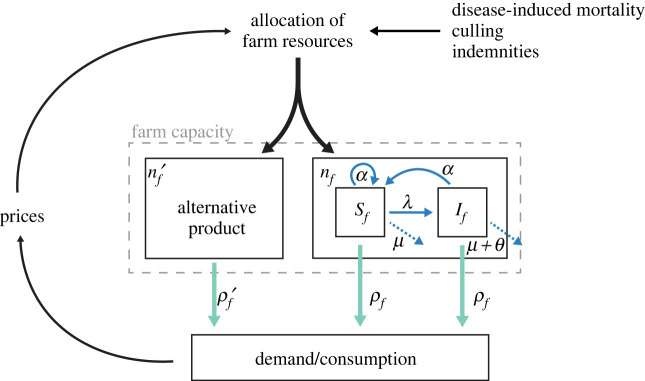
Illustration of the modelled dynamics at the level of households. Households allocate their limited resources to two different production sectors in an optimal way in order to maximize their income. Resource allocation is based on market prices and expected productivity of the livestock herd, which in turn is constrained by losses of animals due to infection. The harvest of animals and the alternative good is displayed with green arrows while biological processes—the demographic and epidemiological dynamics of the animal herd—are shown with blue arrows. The herd is entirely self-renewed, and animals are all born susceptible and can transit to an ‘infected’ category in the course of their life through infection. *n*_*f*_ and *n*_*f*_′ are the number of livestock and alternative product units in the farm, respectively, *S*_*f*_ and *I*_*f*_ are susceptible and infected livestock numbers, respectively, *ρ*_*f*_ and *ρ*_*f*_′ are sale rates of the livestock and alternative product, respectively, *α* is the breeding rate of the livestock species, *μ* is the natural mortality rate, *λ* is the force of infection, *θ* is the disease-induced removal rate of infected animals. The sell rate *ρ*_*f*_ applied by a farmer *f* ensures demographic stability. Therefore, a large proportion of infected animals and/or high rate of departure due to disease (*θI*_*f*_/*n*_*f*_) reduces the proportion of animals available for sale.

The farm dynamics and the profit maximization problem faced by households are illustrated in figure 1. Households’ basic income is derived from the sale of animals and sale of units of the alternative product. This basic profit of households is the product of the number of units produced and market price for each unit. Additionally, farmers may receive indemnities from the state to compensate the losses incurred due to the infectious disease. A farmer *f*’s profit is therefore defined as

2.1 $$U_{f}=\rho_{f}n_{f}P+\rho_{f}^{\prime }n_{f}^{\prime }P^{\prime }+P_{s}\theta I_{f}.$$

The farmer *f* simultaneously adjusts four variables, namely the number *n*_*f*_ of animals on the farm, the number *n*_*f*_′ of units of alternative production, and their sell rates *ρ*_*f*_ and *ρ*_*f*_′. *P* and *P*′ are the market prices, with individual farmers being price-takers (the population of farmers is considered sufficiently large to ensure atomicity of the market). *I*_*f*_ is the number of infected animals, *θ* the mortality rate of infected animals and *P*_*s*_ is the amount of indemnity provided by the state for each dead animal as a result of infection. Production numbers *n*_*f*_ and *n*_*f*_′ both consume household resources, mainly labour and land. Households in smallholder livestock farming systems usually have a limited access to input markets and financial credits. They use livestock farming as a way to derive a profit by using mostly family labour and rarely use the revenue derived from livestock to expand their enterprise by purchasing inputs or hiring labour [29]. Therefore, to keep the economic model as simple as possible, we assume farmers decide on the allocation of a constant amount of resources, mainly composed of farm land and family labour, between the two agricultural productions, and we signal all variables related to the alternative good, where disease dynamics are absent, with a ‘′’ symbol. The resource limit is equal for each farm and is denoted by *M*_*F*_. The constraint implies

2.2 $$M_{F}\geq \omega n_{f}+n_{f}^{\prime }.$$

As stated earlier, the two goods are considered to be perfect substitutes-in-production: any unit decrease in *n*_*f*_ enables farmers to allocate some farm resources to *ω* more units of the alternative product: ∂*n*_*f*_′/∂*n*_*f*_ = −*ω*. All newborn animals are susceptible (*S*_*f*_) to infection and, in the course of their life, can be sold, die, or be infected with the pathogen. Infected animals (*I*_*f*_) can likewise die or be sold, but their mortality rate is higher. The herd dynamics are described by the following set of differential equations:

2.3 $$\begin{array}{rl} \dot{S_{f}} & =\alpha (S_{f}+I_{f})-\mu S_{f}-\rho_{f}S_{f}-\lambda S_{f}\\ \dot{I_{f}} & =\lambda S_{f}-\mu I_{f}-\rho_{f}I_{f}-\theta I_{f}\\ n_{f} & =S_{f}+I_{f} \end{array}\}$$

where *α* is the breeding rate of the livestock species, *μ* is a natural mortality rate (with *μ* < *α*), *λ* is the force of infection (externally determined, in the individual-farm part of the model) which is the rate of new infections per susceptible animal per unit time, and *θ* is the removal rate of infected animals resulting from disease-induced mortality or government-initiated mass culling. To summarize the dynamics, at each time step farmers sell a number of animals *ρ*_*f*_*n*_*f*_ (i.e. a fraction *ρ*_*f*_ of the herd, including susceptibles and infected animals) while a number *μn*_*f*_ of animals (including susceptibles and infected animals) die due to baseline mortality and a number *θI*_*f*_ die as a result of infection. Farmers get a benefit *P* for each animal sold and a monetary compensation *P*_*s*_ from the government for each animal dead as a result of infection. Note that *α* and *μ* are constant in this model, i.e. the breeding and basic mortality rate depend solely on biological properties of animals and their environment. In this model, we assume farmers do not apply a differential treatment to infected and susceptible animals and the same sale rate *ρ*_*f*_ applies to both categories. The sale and consumption of sick animals is frequently observed in livestock systems of low- and middle-income countries [17]. For an alternative model where farmers can destock sick animals at a higher rate, see Delabouglise & Boni [30]. A discussion of our choice of modelling an endemic equilibrium at farm level is provided in electronic supplementary material, S3.

We make three preliminary observations. First, there is a unique positive value of sell rate *ρ*_*f*_ ensuring herd demographic stability (i.e. constant population size) (see electronic supplementary material, S4). The sell rate *ρ*_*f*_ is therefore strictly determined by the external infection pressure *λ* and the severity of disease *θ*. If *λ* = 0 (in a disease-free population) or *θ* = 0 (the disease has no effect on mortality) farmers simply destock at a rate equal to the surplus, i.e. the difference between breeding and mortality rate *ρ*_*f*_ = *α* − *μ*, hereafter referred to as *ρ*_0_. With increasing disease incidence and/or disease severity, farmers reduce *ρ*_*f*_: more adult animals need to be kept for breeding rather than being sold in order to compensate the loss of animals due to disease. All farms in our model have the same properties and face the same force of infection *λ*, thus for an individual farmer we write *ρ*_*f*_ = *ρ*(*λ*) to indicate that the force of infection influences the sell rate (as empirically observed in [17]). The second observation is that, for high values of either *λ* and *θ* the herd goes extinct regardless of the value of *ρ*_*f*_. This outcome is obtained when the excess departure due to disease mortality exceeds the self-renewing capacity of the herd, i.e. when

2.4 $$\lambda \frac{\theta -(\alpha -\mu )}{\theta +\mu }>\alpha -\mu .$$

The third observation is that the expected proportion of infected animals in the herd (*Z*) is directly determined by *λ*

2.5 $$\frac{I_{f}}{n_{f}}=Z(\lambda )=\frac{\lambda }{\lambda +\mu +\theta +\rho (\lambda )}.$$

The sell rate of the alternative good is not affected by the infectious disease. Just like the case of livestock in a disease-free state, it is determined by the biological constraint of this alternative production (the natural population growth rate of the farmed animals or crops). Therefore, the sell rate of alternative product *ρ*_*f*_′ is independent of *λ* and equal to a constant noted *ρ*_0_′. The marginal profit function of herd expansion (adding one more animal to the herd) can be defined as

2.6 $$\frac{\partial U_{f}}{\partial n_{f}}=\rho (\lambda )P+\theta Z(\lambda )P_{s}-\omega \rho_{0}^{\prime }P^{\prime }.$$

*ρ*(*λ*)*P* + *θZ*(*λ*)*P*_*s*_ and *ωρ*′*P*′ are the marginal benefit and marginal opportunity cost, respectively, of livestock herd expansion. By adding one livestock head to their herd, the farmers forego the opportunity of producing *ω* units of alternative product. The optimal livestock herd size is therefore determined by market prices *P* and *P*′, the level of losses of livestock incurred, which is captured by *ρ*(*λ*), and the revenue earned from state indemnities, which is captured by *θZ*(*λ*)*P*_*s*_.

### 2.3. Disease dynamics in a population of farms

In addition to the individual farm model, we track the total number of susceptible ($S=\sum_{\;f}S_{f}$) and infected ($I=\sum_{\;f}I_{f}$) animals in a region or community with many farms. Each individual animal is exposed to a risk of pathogen transmission through direct or indirect contacts with animals of the same farm or different farms. Indirect or direct infectious contacts between animals located in different farms occur frequently and in different ways in smallholder farming systems where biosecurity and biocontainment measures are very limited. Examples of such transmission pathways include: (i) farmers or roaming animals crossing neighbouring farms and carrying pathogens on them [13,31], (ii) domestic animals of different farms mixing in communal grazing areas [32,33], (iii) feed consumed by animals contaminated with viscera or faeces of infected animals from other farms [13,14,34,35], (iv) live animal traders visiting different farms and potentially carrying pathogens (on their clothes or vehicles), and (v) sale of finished animals in livestock markets, where cross-contamination is also likely [32,34–37]. For the sake of mathematical tractability, we assume homogeneous mixing of the animal population and do not differentiate intra-farm and inter-farm transmission rates. For some of the livestock epidemic diseases causing the highest production losses, including Newcastle disease in poultry, ASF in pigs and PPR in small ruminants, horizontal transmission between live animals is the predominant transmission pathway and vertical transmission—infected females giving birth to live infected offspring—has not been documented or remains dubious [38–40]. Therefore, we only consider horizontal disease transmission here. The resulting transmission model involves classical dynamics that are nonlinear and dependent on the total number of infected animals, and they determine the community-level force of infection *λ* that is experienced by individual animals.

2.7 $$\begin{array}{rl} \dot{S} & =\alpha (S+I)-\mu S-\rho S-\beta SI\\ \dot{I} & =\beta SI-\mu I-\rho I-\theta I\\ n & =S+I \end{array}\}$$

with *β* as the community-level transmission parameter. The force of infection *λ* experienced by a single farm is

2.8 λ=βI=βn−(μ+θ+ρ).

And the reproduction ratio of the disease, the number of infections caused by a single infected animals in a fully susceptible population, is

2.9 $$R_{0}=\frac{\beta n}{\mu +\theta +\rho }.$$

Transmission is density-dependent, i.e. the reproduction ratio is positively correlated with the size of the animal population and the establishment of the disease in the population is only possible if the animal population *n* is above a given threshold *n*_*T*_, which results in *R*_0_ > 1. Indeed, for diseases transmitted by direct contacts between animals or by exchange of contaminated material between farms, disease transmission is likely to be facilitated by a high proximity between animals and between animal farms. Therefore, a high number of animals in a given geographical area promotes disease transmission.

### 2.4. Livestock meat price

We consider, without loss of generality, that the slaughter of an animal produces one unit of meat so that the total quantity of meat (*q*) and alternative product (*q*′) produced by the population of farmers at a given time are

2.10 q=ρn

and

2.11 $$q^{\prime }=\rho^{\prime }n^{\prime }.$$

The meat obtained from slaughtered animals is sold to the consumers of the region at a given domestic price determined by the level of meat supply and consumer demand. A consumer chooses the quantity of livestock meat *q* and alternative product *q*′ purchased based on two criteria: price and perceived quality, the latter being affected by the presence of the disease in the consumed animal. For the sake of mathematical tractability, we assume a linear meat demand model. The domestic prices *P* and *P*′ are bounded between 0 and maximum values *P*_max_ and *P*′_max_ which correspond either to price ceilings enforced by the authorities or to the import meat prices (i.e. if *P* reaches *P*_max_, consumers will turn to imported meat). Hence the expressions of *P* and *P*′ are

2.12 $$\begin{array}{rl} P & =a-bq-cq^{\prime }-dZ\;\mathrm{if}\;0<a-bq-cq^{\prime }-dZ<P_{\max}\\ & =0\;\;\;\;\mathrm{if}\;a-bq-cq^{\prime }-dZ<0\\ & =P_{\max}\;\;\;\;\mathrm{if}\;a-bq-cq^{\prime }-dZ>P_{\max} \end{array}\}$$

and

2.13 $$\begin{array}{rl} P^{\prime } & =a^{\prime }-b^{\prime }q^{\prime }-cq\;\mathrm{if}\;0<a^{\prime }-b^{\prime }q^{\prime }-cq<P_{\max}^{\prime }\\ & =0\;\;\;\mathrm{if}\;a^{\prime }-b^{\prime }q^{\prime }-cq<0\\ & =P_{\max}^{\prime }\;\;\;\mathrm{if}\;a^{\prime }-b^{\prime }q^{\prime }-cq>P_{\max}^{\prime }. \end{array}\}$$

With *a*, *a*′, *b*, *b*′, *c* and *d* constant positive parameters and *Z* the risk that the purchased meat come from an infected animal. See electronic supplementary material, S5 for a detailed description of the demand model and price formation. A high value of *b* indicates that consumers are inflexible in their consumption of livestock meat and the quantity of livestock meat consumed is not highly responsive to changes in meat price *P*. The parameter *c* relates to the potential substitution effects between the consumption of the two products. The parameter *d* is the disutility associated with the consumption of a unit of meat originating from an infected animal. In order to test the robustness of our results to the nature of the demand model, we simulated the system response with a log-linear meat demand model with constant own-price elasticity (see electronic supplementary material, S6).

While the farm production model assumes a linear relationship between the production of animals and alternative goods, the concavity of the consumers’ utility function of the consumption of the two products (see electronic supplementary material, S5) results in a single disease-free equilibrium (DFE) level of production. The reader is referred to electronic supplementary material, S7 for the details of the computation of the Nash equilibria.

## 3. Results

Our economic-epidemiological livestock model where individual farms simultaneously produce goods and disease risk admits a maximum of three Nash equilibria, two stable and one unstable (see proof in electronic supplementary material, S7). Specifically, the system has either a single stable Nash equilibrium, or it has three Nash equilibria (two stable, one unstable).

### 3.1. Stability of the disease-free equilibrium

In the absence of the disease, the herd sell rate *ρ* is constant and equal to *ρ*_0_. Marginal benefit of herd expansion is a linear decreasing function of the total population *n*, which results in a single DFE combination of market prices and population sizes {*P*_DFE_, *P*′_DFE_, *n*_DFE_, *n*′_DFE_} (see mathematical formulation of equilibria in electronic supplementary material, S7).

The food demand model is governed by the degree of inflexibility of consumers regarding the quantity of products they consume—described by the two coefficients *b* and *b*^′^—and the extent to which the two products can be substituted—described by the coefficient *c*. The own-price elasticities at disease-free market equilibrium are

3.1 $$\begin{array}{rl} \epsilon & =\frac{\partial q}{\partial P}\frac{P_{\mathrm{DFE}}}{q_{\mathrm{DFE}}}=-\frac{P_{\mathrm{DFE}}}{\rho_{0}n_{\mathrm{DFE}}}\frac{b^{\prime }}{bb^{\prime }-c^{2}}\\ \;\mathrm{and}\;\epsilon^{\prime } & =\frac{\partial q^{\prime }}{\partial P^{\prime }}\frac{P_{\mathrm{DFE}}^{\prime }}{q_{\mathrm{DFE}}^{\prime }}=-\frac{P_{\mathrm{DFE}}^{\prime }}{\rho_{0}^{\prime }n_{\mathrm{DFE}}^{\prime }}\frac{b}{bb^{\prime }-c^{2}} \end{array}\}$$

Similarly, we can derive an expression of the cross-price elasticities of demand for the livestock and alternative product at DFE

3.2 $$\begin{array}{rl} \epsilon_{c} & =\frac{\partial q}{\partial P^{\prime }}\frac{P_{\mathrm{DFE}}^{\prime }}{q_{\mathrm{DFE}}}=\frac{P_{\mathrm{DFE}}^{\prime }}{\rho_{0}n_{\mathrm{DFE}}}\frac{c}{bb^{\prime }-c^{2}}\\ \;\mathrm{and}\;\epsilon_{c}^{\prime } & =\frac{\partial q^{\prime }}{\partial P}\frac{P_{\mathrm{DFE}}}{q_{\mathrm{DFE}}^{\prime }}=\frac{P_{\mathrm{DFE}}}{\rho_{0}^{\prime }n_{\mathrm{DFE}}^{\prime }}\frac{c}{bb^{\prime }-c^{2}} \end{array}\}$$

where high values of *b* and *b*′ are indicative of inelastic demand for the livestock meat and the alternative product, respectively. A high value of *c* is indicative of a high cross-price elasticity between the two alternative products: with a higher value of *c*, more consumers are likely to increase their demand for one product in response to an increase of the price of the other. The price response to a change in production of livestock—the increase of livestock price resulting from a decrease in livestock production—is an inverse function of the own-price elasticity: in the case of inelastic demand (low own-price elasticity) any change in the livestock supply will result in a comparatively higher degree of price shift.

The stability of the DFE depends on *R*_0_(*n*_DFE_). If *R*_0_(*n*_DFE_) < 1 the disease cannot persist in the population and any introduced pathogen is ultimately eradicated in the population. If *R*_0_(*n*_DFE_) > 1, the population can be invaded by the disease, in which case livestock production and consumer demand are altered. Analytically, we can show that the DFE is unstable if and only if

3.3 $$n_{\mathrm{DFE}}>\frac{\alpha +\theta }{\beta }=n_{T}$$

as in traditional epidemiological models; this corresponds to a situation where the community’s total population size is above a threshold value *n*_*T*_ allowing sustained disease transmission. We observe that a sufficiently large disease-induced mortality rate (*θ* in our model) reduces disease transmission and enables the stabilization of a DFE, as it shortens the time period during which infected animals can transmit the disease. We denote *θ*_*T*_ as the threshold value of *θ* above which the DFE is stable (see a mathematical formulation of *θ*_*T*_ in electronic supplementary material, S7). Also, a high breeding rate (*α*) has the same effect as a high disease-induced mortality (*θ*), since it implies that farms have rapid turnover in their animal populations, and the pathogen is not able to invade or establish; this effect is not present in human disease models.

### 3.2. Stability of the endemic equilibrium

**Figure 2.**
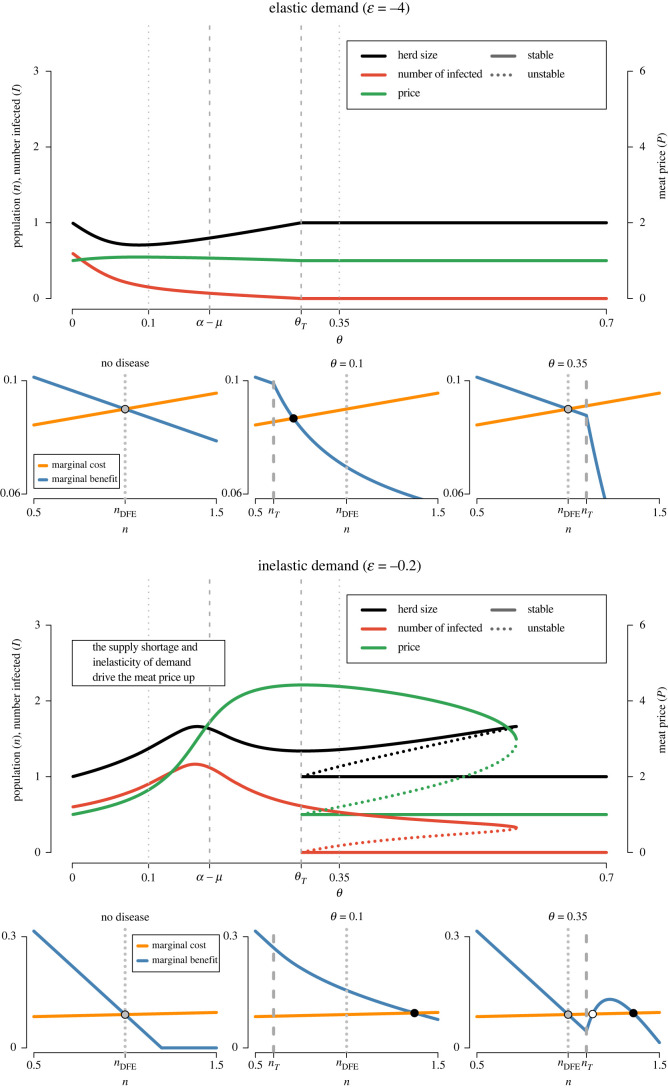
Effect of the level of disease-induced removal rate (*θ*) on the system Nash equilibria population sizes (*n*), the numbers of infected animals per herd (*I*) and livestock market prices (*P*). All variables are scaled to be simultaneously represented on the same graph. Stable and unstable equilibria are represented with solid and dotted curves, respectively. The relationship is explored for own-price elastic (low *b*, top graph) and own-price inelastic (high *b*, bottom graph) demand for livestock meat. In both cases, the own-price elasticity of the demand for the alternative product is high (low *b*′, $\epsilon^{\prime }=-4$), and there is no substitution effect between the consumption of the two products (*c* = 0), no food scare (*d* = 0) and no state indemnities (*P*_*s*_ = 0). See electronic supplementary material, figure S3 for an alternative setting with inelastic demand for the alternative product. Additionally, three smaller graphs display the relationship between the marginal benefit (blue curve) and marginal opportunity cost (orange curve) of expanding one’s herd size (*n*_*f*_) for households in response to increasing population size (*n*) under three conditions: no infectious disease, disease with low disease-induced departure rate (*θ* = 0.1) and disease with high disease-induced departure rate (*θ* = 0.35). Dots indicate Nash equilibria which are either disease-free and stable (grey dots), endemic stable (black dots) or endemic unstable (white dot). Dotted lines and dashed lines indicate disease-free equilibrium herd size (*n*_DFE_) and threshold population size enabling disease propagation (*n*_*T*_), respectively.

At endemic equilibrium, provided the disease causes some mortality (*θ* > 0), the sell rate *ρ* is affected by the infection risk of animals (*λ* in the model) which, in turn, is determined by the population size *n*, since the disease prevalence is increased by the population size. In the face of increasing risk of mortality due to disease, farmers have to lower their sell rate in order to maintain the balance between births and departures. Consequently, the marginal return of herd expansion ∂*U*_*f*_/∂*n*_*f*_ is no longer a linear decreasing function of the community size *n*. The sign of ∂*U*_*f*_/∂*n*_*f*_ is determined by a continuous cubic function of *n*, shown in electronic supplementary material, S7. For very large *n*, ∂*U*_*f*_/∂*n*_*f*_ is ultimately negative, ensuring that in the absence of DFE, there is at least one endemic equilibrium (as illustrated in figure 2).

This also implies that, under some specific conditions, for a range of intermediate values of *n*, the marginal return of herd expansion ∂*U*_*f*_/∂*n*_*f*_ can be an increasing function of *n*, i.e. farmers make a higher profit from expanding their herd when the community size *n* is larger, creating a positive feedback loop between community size and incentive to stock more animals. Qualitatively, we observe that this counterintuitive outcome is obtained when the demand for livestock meat is inelastic and the disease-induced mortality is high (low ϵ, high *θ*). Under this condition, when the community size *n* is moderate but sufficient to sustain disease transmission, a limited increase in community size paradoxically results in a reduction in meat production and an increase of meat market price *P*, as the steep increase in the proportion of infected animals compels farmers to considerably reduce their sell rate. The positive relationship between ∂*U*_*f*_/∂*n*_*f*_ and population size allows the establishment of three Nash equilibria, as illustrated in figure 2 (bottom right graph). The intermediate equilibrium, characterized by ∂^2^*U*_*f*_/∂*n*∂*n*_*f*_ > 0, is unstable. In this situation of system bistability, farmers have two stable strategies: (i) keeping a low herd size ensuring disease control (*R*_0_ < 1), good productivity (*ρ* = *ρ*_0_) and moderate sale price (*P* = *P*_DFE_), or (ii) keeping a large herd size enabling disease transmission (*R*_0_ > 1) with productivity undermined by disease losses (*ρ* < *ρ*_0_), which are compensated for by high sale price (*P* > *P*_DFE_).

This result was also obtained with a log-linear meat demand model with constant own-price elasticity, by using a simulation procedure (see electronic supplementary material, S6).

### 3.3. Effect of disease-induced livestock mortality on herd size

The effect of disease-induced mortality on the animal population is strongly dependent on the elasticities of demand for livestock meat and the alternative product (ϵ and $\epsilon^{\prime }$, related to *b* and *b*′). Under endemic equilibrium, a high disease-induced mortality rate forces farmers to sell fewer animals. If the demand for livestock meat is elastic, the shortage of livestock supply induces a limited increase of livestock price. The limited price increase does not compensate for a farmer’s production loss, and farmers would tend to reduce their herd size in order to redirect their resources to the production of the alternative good; this in turn reduces disease prevalence (figure 2, top). However, if demand is sufficiently inelastic, then the shortage of livestock due to disease-induced mortality induces a comparatively high price increase. This price increase offsets the production loss and farmers expand their herd to increase profits from the high livestock meat sale price, which in turn increases disease prevalence (figure 2, bottom). Note that the relationship between herd size and disease-induced mortality is nonlinear, with a peak in animal population when *θ* is close to the livestock natural growth rate *α* − *μ*.

When *θ* < *θ*_*T*_ the endemic equilibrium is stable. When *θ* > *θ*_*T*_ the DFE is stable. The equilibrium is unique in the case of elastic demand for livestock meat, as there is no benefit for farmers in maintaining a herd size above *n*_*T*_ (figure 2, top). When livestock meat demand is inelastic, bistability may occur, with two simultaneously stable equilibria (one endemic, the other disease-free; figure 2). At the endemic equilibrium, herds are kept sufficiently large to allow the disease to circulate. The high mortality of livestock due to disease forces farmers to limit the sale of their animals, but this loss is compensated for by the high meat market prices, providing sufficient incentive for farmers to maintain large herds. One equilibrium is clearly socially optimal (the DFE) while the second (the endemic equilibrium) is characterized by ongoing disease circulation, low consumer welfare due to high meat prices, and inefficient use of farm resources.

As illustrated in the electronic supplementary material, figure S3, inelastic demand for the alternative product considerably reduces variation of animal population size. The reason is that any expansion of the livestock population results in a decreased production of alternative good. If the demand for the alternative good is inelastic, the resulting increase in the alternative good’s price *P*′, encourages farmers to rebalance their resource allocation by maintaining a high production of the alternative good and limit livestock herd expansion. Likewise, when livestock herd size is reduced and production of the alternative good is increased, the resulting drop in *P*′ encourages farmers to rebalance by shifting resources back into livestock production. As a consequence, bistability is less likely: the range of values of *θ* allowing both disease-free and endemic equilibria is smaller.

### 3.4. Effect of demand structure on farmers’ strategic response and policy implications

**Figure 3.**
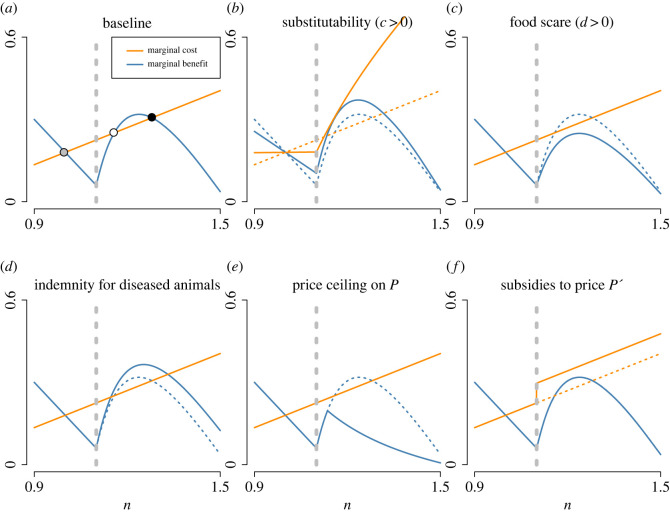
Illustration of the effect of some variations in demand structure and policy intervention on bistability of disease-free (grey circle) and endemic stable (black circle) Nash equilibria. Lines indicate baseline (dashed) and new (solid) marginal benefits (blue) and marginal costs (orange) of herd expansion for increasing values of the community size *n*. (*a*) Baseline scenario with inelastic consumer demand. Effects of changes in the demand structure include (*b*) the introduction of a substitution term between the two products supplied by farmers (*c* > 0), (*c*) aversion of consumers towards consumption of meat from diseased animals (*d* > 0). Policy interventions include (*d*) the payment of an indemnity to farmers for each dead animal as a result of the disease (*P*_*s*_ > 0), (*e*) enforcement of a low ceiling on livestock market price (lower *P*_max_) through regulation or through decreasing tax on meat import, and (*f*) a subsidy to the sale price *P*′ of the alternative product. The vertical dashed grey line is the threshold community size enabling sustained disease circulation (*n*_*T*_). All parameters *a*, *a*′, *b* and *b*′ are set so that *n*_DFE_ = 1, *P*_DFE_ = 1 and *P*′_DFE_ = 1. The other parameters are $\alpha =0.2,\mu =0.02,\theta =0.35,\beta =0.5,\epsilon^{\prime }=-0.4$ and *P*_max_ = *P*′_max_ = 4.

The results in the previous section show that inelastic demand for livestock products can exacerbate livestock epidemics and undermine efforts aimed at eradicating an infectious disease. If a government initiates a control programme based on the rapid detection and culling of infected animals or animals located in infected farms, which is equivalent to an increase of disease-induced mortality, we see that an expansion of herd size is a stable strategic response for farmers, and that this strategy allows for persistence of the disease at levels of culling which would normally result in disease eradication. This scenario corresponds to the bistability shown in figure 2, bottom right and shown in figure 3*a*. Similarly, this strategic response may allow a highly pathogenic infectious agent to maintain its circulation in a livestock population when eradication would be expected if the farmers did not increase their herd size. In this section, we explore different modifications to the demand structure and policy interventions which may solve this perverse effect.

Introducing a substitution term between the consumption of the two products (*c* > 0) or a consumer’s aversion for livestock disease (*d* > 0) both decrease the likelihood of bistability. When the two products are substitutes, the drop in livestock production caused by the disease results in a subsequent increase in the alternative product’s price, as consumers partly compensate for decreased livestock meat with an increased demand for the alternative product, thereby increasing the marginal opportunity cost of herd expansion when the disease circulates (figure 3*b*). Consumer’s aversion towards meat originating from infected livestock (the ‘food scare’ effect) enables a decrease of the endemic equilibrium livestock meat price and thereby decreases the marginal benefit of livestock herd expansion at endemic equilibrium (figure 3*c*).

Introducing the payment of an indemnity for each animal lost by farmers as a result of the disease (*P*_*s*_ > 0) worsens the situation, as it increases the marginal benefit of herd expansion when the disease is circulating (figure 3*d*): farmers are willing to stock more animals not only because of the high sale price of the survivors, but also because disease losses are compensated by state indemnities. On the contrary, enforcing a low ceiling on livestock meat market price *P*_max_, through price control or a decrease of meat import taxes, sets a limit to the marginal benefit farmers derive from expanding their herd and thereby reduces the likelihood of endemic equilibrium (figure 3*e*). Finally, a subsidy on the sale price of the alternative product in the case of endemicity (subsidies are only granted if the disease circulates) has positive effects, as it increases the marginal benefit of production of the other good and, therefore, the marginal opportunity cost of livestock herd expansion (figure 3*f*).

## 4. Discussion

In most countries, livestock meat is a necessary good characterized by inelastic demand. In general, meats from different animal species are consumption substitutes with positive cross-price elasticities [41–44]. Supply shocks caused by livestock epidemics tend to drive livestock meat prices up. This is exemplified by the recent ASF epidemic in China which has led to a fall in pig production of nearly 50%, resulting in a more than twofold increase in the price of pork [45]. Despite the risk of disease-induced production losses, a USDA report indicated that farmers were willing to restock early or expand their herd after the onset of the ASF epidemic, in anticipation of the benefits obtained from the sale of surviving animals [46]. An economic analysis confirmed that the benefits obtained by the Chinese pork industry as a result of the supply shocks exceeded the costs resulting from production losses, with up to 40% excess returns compared with baseline predictions during the 2019 spring festival, when the demand for pork was highest [47–49]. Increasing net profits seemed to encourage the rapid rebuilding of pig breeding populations, and production increased faster than expected [50]. However, this expansion of pig farms was unequally distributed across production systems. Although accurate official data are missing, reports indicate a large fraction of Chinese small-scale farms interrupted or quit pork production while large-scale farms rapidly expanded in number and size [48,51]. This concentration of the pig industry is partly due to (i) governmental support (e.g. subsidies, loans) benefiting mostly, if not exclusively, large-scale producers, and (ii) a relaxation of environmental regulations and land-use restrictions, while small-scale farms were banned from restocking in some provinces [47,48]. Owing to economies of scale and easy access to financial credit, large-scale farms are also more likely to implement preventive actions to limit production losses due to infectious diseases, giving them a competitive advantage over small-scale producers. Our model does not capture such dynamics, as we did not incorporate heterogeneity in farm sizes and costs of risk-reduction measures. However, it provides some indications on the potential joint dynamics of the pig population and ASF prevalence that will unfold on the long run.

The co-occurrence of infectious disease and market dynamics can be observed with other types of animal products. The recent mass culling of farmed minks in Europe resulted in a supply shock on the global fur market. Consequently, even though the farming of minks and other wild animals are among the suspected drivers of the emergence of SARS-CoV-2 in humans [52,53], the Chinese mink industry currently benefits from the rise in fur prices [54].

When disease spread is facilitated by a high density of animals, the most critical aspects of disease control are the evolution of the size of the livestock population in response to epidemics, their resulting control policies, and their market effects. Our model shows that in the context of inelastic demand for meat of the affected species, the adaptation of herd size by farmers can have some counterintuitive outcomes and undermine the benefits of a disease control policy based on the culling of affected herds. This work complements other theoretical studies of farmers’ responses to livestock diseases and disease control policies. Using a principal-agent framework, Gramig *et al.* studied the adverse impact of indemnity payments to farms affected by diseases on investments in farm biosecurity and truthful disclosure of disease outbreaks [9,55]. Beach *et al.* also studied the effect of different incentives on the uptake of biosecurity measures specifically for avian influenza [8]. More recently, Horan *et al.* demonstrated that livestock diseases can, contrary to the common expectations, stimulate the trade of live animals between two regions specialized in the breeding or feeding of ruminants [56]. Another study showed the potential perverse effect of culling policies when the indemnities provided to farmers encourage an expansion of their farm size [57], which can be considered as an example of the cobra effect. Here, we demonstrate that, even without indemnities, the market response alone may drive an overstocking of animals with adverse epidemiological and economic effects, and that inelastic demand makes this cobra effect more likely.

The policy messages we derive from our analysis are twofold. First, in the presence of inelastic demand for livestock meat, animal disease control policies should be complemented with economic measures aimed at discouraging an expansion of the herds of susceptible animals. One option is to limit livestock meat price increase. While enforcing a ceiling on market price may be impracticable, a temporary removal of entry barriers to imports of livestock products, enabling the supply of the domestic market with imported meat, can contribute to maintaining domestic prices at a low level. Our results show that state indemnities provided to farmers to compensate the loss of animals due to disease have perverse effects, but removing these subsidies would not be acceptable for farmers confronted with large income losses, especially if these losses are due to depopulation measures initiated by the government. A potential solution would be to encourage farmers to invest in an alternative production, by conditioning a fraction of the state indemnities on the stocking of an alternative species of livestock, preferentially a close consumption substitute to the species affected by the disease. In the case of ASF in China, a recent study underlined the benefits of subsidizing poultry production as complementary to supporting the pig industry, as poultry is a close consumption substitute to pigs and has the added advantages of (i) being less resource-consuming than pork/beef production, and (ii) rapidly available to consumers because of its short production cycle [45]. Second, livestock disease control benefits from diversification of protein production and consumption, which policymakers can favour by making more substitute product available. The coexistence of different types of food productions satisfying the protein needs of consumers—based on livestock, fish, insects, or vegetables—increases the elasticity of demand and limits market price response to potential supply shocks caused by epidemics. In other words, consumers and producers respond to epidemics by replacing susceptible animals with their substitutes rather than increasing their expenditure in the purchase of susceptible livestock produced in lower quantity. Excessive stocking of susceptible animals can also be averted if information made available to consumers on ongoing disease outbreaks lessens their demand for meat of the susceptible species, especially if the disease has some zoonotic potential. However, this approach may add complex future price–disease dynamics, as food scares can have long-lasting and unpredictable effects on patterns of meat consumption which can outlive their originating epidemics [24].

The main limitation of our model is that it only captures one aspect of farmers’ adaptation to disease risk (adjustment of herd size) leaving aside other aspects such as vaccination, disinfection and rapid sale or slaughter of sick animals [17]. The rapid sale of sick animals can be considered as a farmer-initiated increase of the removal rate associated with infection to avoid feeding animals which are likely to die before maturity. Our model is steady state in nature, while the emergence of a new disease or an outbreak caused by the introduction of a pathogen in a new region are inherently dynamic processes involving rapid and large discrete changes in biological and economic conditions. The results must therefore be interpreted as an indication of the long-term evolution of the system resulting from such shocks, including establishment or not of pathogen endemicity and resulting socio-economic consequences. A dynamic epi-economic model would be a natural extension of this study. The results are based on the assumption that the transmission of the disease is to some extent density-dependent, i.e. that its spread is facilitated by a high population size. This assumption holds for most cases of infectious disease transmitted through direct contact within and between herds, but it may not be relevant to all epidemiological settings. For example, when disease transmission is mainly mediated by long-distance trade movements, disease transmission may be dependent on the frequency of animal transports and movement network properties rather than on the number of farmed animals [30]. Finally, the farm economic model used here assumes a static farm resource amount distributed across several agricultural products, which we believe is consistent with most smallholder farming systems. However, allowing an increase in farm resources, with a reallocation of the revenue earned or the use of credits to expand the farm enterprise—through the purchase of additional land or inputs or the hiring of farm workers—would be an interesting extension of the model. Moreover, it would allow for the exploration of additional regulatory instruments, like the allocation of farm credits to encourage the production of alternative goods by farmers in the case of epidemics.

With a consistently high frequency of disease emergence observed at the interface between animals and humans and rising concerns around the environmental impact of the livestock industry, it is increasingly clear that prospects for the development of livestock production systems are tightly linked to their ability to reduce the burden of infectious disease and effectively manage the risk of livestock epidemics. Our analysis underlines some potential effects of strategic reactions of farmers to epidemics, with joint economic and epidemiological dynamics potentially leading to an overstocking of animals. This adverse outcome may be corrected in the short term by providing the right incentives to farmers, and in the long term by favouring diversification of protein production systems.

## Data Availability

The original study dataset used to draw the electronic supplementary material, figure S1 is available at https://osf.io/ws3vu/. The data are provided in electronic supplementary material [58].
